# Stimuli-responsive drug delivery systems for head and neck cancer therapy

**DOI:** 10.1080/10717544.2021.1876182

**Published:** 2021-01-27

**Authors:** Jingou Liang, Bina Yang, Xuedong Zhou, Qi Han, Jing Zou, Lei Cheng

**Affiliations:** a State Key Laboratory of Oral Diseases, National Clinical Research Center for Oral Diseases, West China School of Stomatology, Sichuan University, Chengdu, China; b Department of Pediatric Dentistry, West China School of Stomatology, Sichuan University, Chengdu, China; c Department of Cariology and Endodontics, West China School of Stomatology, Sichuan University, Chengdu, China

**Keywords:** Stimuli-responsive drug delivery systems, head and neck cancer, intelligent biomaterials, anticancer therapy, tumor microenvironment

## Abstract

Head and neck cancer (HNC) is among the most common malignancy that has a profound impact on human health and life quality. The treatment for HNC, especially for the advanced cancer is stage-dependent and in need of combined therapies. Various forms of adjuvant treatments such as chemotherapy, phototherapy, hyperthermia, gene therapy have been included in the HNC therapy. However, there are still restrictions with traditional administration such as limited in situ therapeutic effect, systemic toxicity, drug resistance, etc. In recent years, stimuli-responsive drug delivery systems (DDSs) have attracted the great attention in HNC therapy. These intelligent DDSs could respond to unique tumor microenvironment, external triggers or dual/multi stimulus with more specific drug delivery and release, leading to enhanced treatment efficiency and less reduced side effects. In this article, recent studies on stimuli-responsive DDSs for HNC therapy were summarized, which could respond to endogenous and exogenous triggers including pH, matrix metalloproteinases (MMPs), reactive oxygen species (ROS), redox condition, light, magnetic field and multi stimuli. Their therapeutic remarks, current limits and future prospect for these intelligent DDSs were discussed. Furthermore, multifunctional stimuli-responsive DDSs have also been reviewed. With the modification of drug carriers or co-loading with therapeutic agents. Those intelligent DDSs showed more biofunctions such as combined therapeutic effects or integration of diagnosis and treatment for HNC. It is believed that stimuli-responsive drug delivery systems showed great potential for future clinic translation and application for the treatment of HNC.

## Introduction

Head and neck cancer (HNC) refers to epithelial malignancies of the paranasal sinuses, nasal cavity, oral cavity, pharynx, and larynx. The high incidence and prevalence of HNC profoundly influence human health and reduce the quality of life (Mehanna et al., 2011). In the 2017 global burden of disease study, around 678,900 incident cases were reported. According to the report from GLOBOCAN, around 450,000 estimated new cases of oral cavity and pharyngeal cancer and 23,000 estimated deaths were reported worldwide in 2018 (Fitzmaurice, 2018). Squamous cell carcinoma (SCC) is the most frequently diagnosed HNC. Previous researches have shown that the risk factors of HNC include tobacco intake, heavy alcohol consumption, human papillomavirus (HPV), betel quid, and additional factors such as certain microorganisms and environmental pollutants (Marur & Forastiere, 2016). The current management of HNC is stage-dependent and based on the multidisciplinary teamwork. Especially, Stage III or IV HNCs need the combination of surgical treatment and other forms of adjuvant treatment such as chemotherapy, phototherapy, hyperthermia, gene therapy, etc. (Feller & Lemmer, 2012; Chi et al., 2015). However, there are still some problems remains unsolved, including low tissue selectivity, poor drug solubility, unfavorable bioavailability, chemical instability and systemic adverse side effects of traditional drug administration (Koo et al., 2005; Godin et al., 2011; Epstein et al., 2012; Gong et al., 2012). Besides, drug resistance and cancer recurrence have become great challenges during the clinical application. Even worse, multidrug resistance (MDR) has been reported in many cases of HNC, which leads to the recurrence and progression of HNC (Pérez-Sayáns et al., 2010).

Drug delivery systems (DDSs) that could transport and release therapeutic agents to the tumor site have provide a promising strategy for the HNC therapy, which are usually consist of carriers and associated therapeutics for improved drug stability, local drug administration and controlled drug release (Davoodi et al., 2018). Previous studies have reported that drug could be transported by DDSs with the enhanced permeability and retention (EPR) effect or active targeting properties. They have been widely explored in the treatment of cancer for the higher curative efficiency and less side effect (Matsumura & Maeda, 1986; Greish, 2010; Wu & Zhou, 2015). In recent years, with the prosperous development of intelligent biomaterials, novel generation of DDSs with stimuli-responsive properties have drawn great attention in the HNC therapy (Kalaydina et al., 2018; Ketabat et al., 2019). Such intelligent DDSs could respond to chemical, physical or biological stimuli like pH, enzymes, light, magnetic field and so on, leading to more precise drug delivery, enhanced tumor penetration, better biocompatibility and more controllable drug release (Zhou et al., 2018; Qiao et al., 2019). Various kinds of stimuli-responsive DDSs such as micelles (Zhou et al., 2018), liposomes (Olusanya et al., 2018), hydrogels (Hajebi et al., 2019) and nanoparticles (Hossen et al., 2019) have been fabricated based on the biocompatible materials that could undergo structural changes including the protonation of specific groups, the cleavage of chemical bonds, the molecular conformational change, etc. in response to endogenous or exogenous stimuli, resulting in controlled drug delivery. With the development of the HNC studies, researchers have found that there occurs a unique tumor microenvironment (TME) during the progression of cancer including low pH, overexpressed specific enzymes, high levels of reactive oxygen species (ROS), upregulation of antioxidant and so on, which provide the opportunity for the application of internal stimuli responsive DDSs for more specific drug delivery and release (Koontongkaew, 2013; Weinberg et al., 2019). The stimuli-responsive DDSs are commonly researched for chemotherapy in the HNC research. Many types of chemotherapeutic drugs have been reported loaded on the intelligent DDSs for improved antitumor effect in recent years (Lee, Lee, Kim, et al., 2014; He et al., 2015; Wei et al., 2018). Gene therapy is another promising therapeutic modality for HNC treatment (Bali et al., 2013). In the DDSs research, gene materials could be transferred to tumor cells leading to cell death and inhibition of tumor growth (Liu, Xue, et al., 2014; Zhang, 2015; Lin et al., 2017). In recent years, more and more researches fabricated novel stimuli-responsive DDSs for gene delivery, particularly co-delivery with other antitumor agents (Ma, 2014; Wang et al., 2016). Moreover, during some other kinds of HNC therapy with exogenous stimulus, physical external stimuli responsive DDSs have also been utilized. In the phototherapy research for HNC, light-responsive therapies such as photodynamic therapy (PDT) and photothermal therapy (PTT) have been widely reported with significant *in situ* therapeutic effect (Fan, Zhu, et al., 2020). In other therapies like hyperthermia for HNC, stimuli-responsive DDSs have also drawn increasing attention. For instance, some magnetic field responsive DDSs have been reported to exhibit *in situ* heat generation and antitumor effect (Su et al., 2019).

Furthermore, the stimuli-responsive DDSs could also be modified, yielding multifunctional DDSs, for active drug delivery, integration of diagnosis and treatment, combined therapy and so on. For instance, for enhanced delivery efficient and specificity, increasing number of researchers developed DDSs with active targeting properties rather than that with EPR effect. Ligand/receptor-mediated endocytosis is among the most used strategies. The DDSs carriers could be linked with various kinds of ligands which could recognize specific receptors on tumor cells, thus leading to improved internalization to tumor cells and cell killing effect (Liu, Gao, et al., 2014; Li, Wen, You, et al., 2016; Nam et al., 2018). Some magnet-based DDSs or DDSs co-loading with fluorescent materials have been designed for theranostic applications which could combine cancer imaging with treatment (Kim et al., 2013; Bhana et al., 2015; Haedicke et al., 2015). Besides, co-delivery of antitumor agents such as chemotherapeutic drugs, photosensitizers (PSs) and gene materials could demonstrate synergistic therapeutic effect and reduced side effect, which showed great research potential recently (Ma, 2014; Miao et al., 2014; Wang et al., 2016).

**Figure 1.**
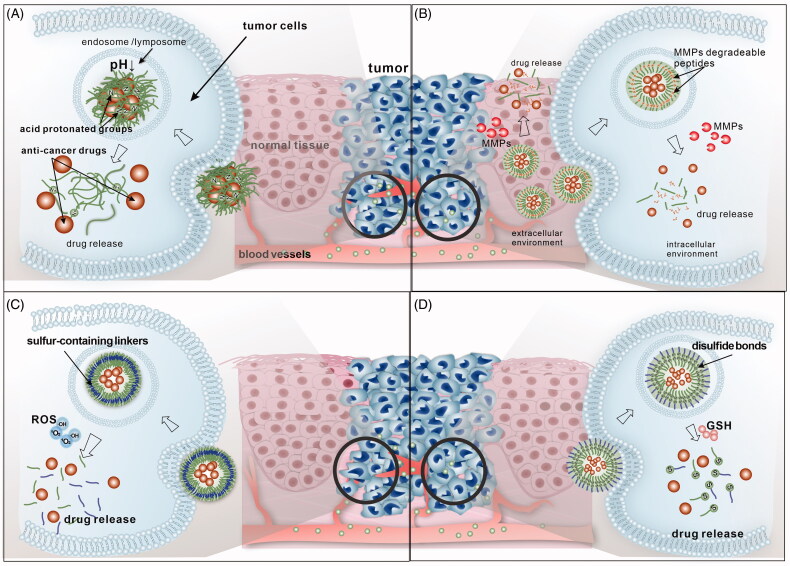
Illustration of internal stimuli responsive DDSs commonly applied for HNC therapy in recent research. (A) PH-responsive DDSs; (B) MMPs-responsive DDSs; (C) ROS-responsive DDSs; (D) redox-responsive DDSs.

**Figure 2.**
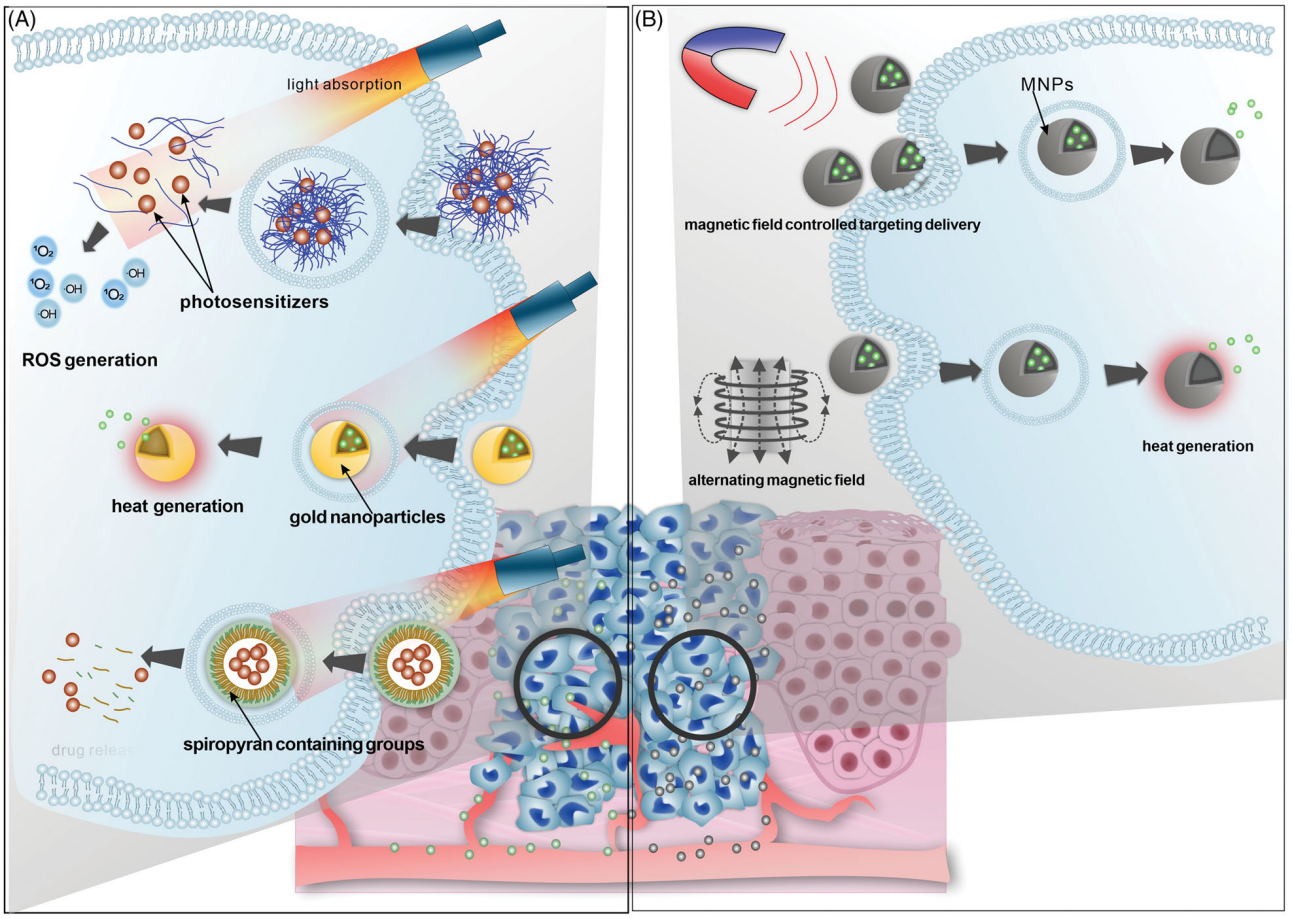
Illustration of external stimuli responsive DDSs commonly applied for HNC therapy in recent research. (A) Light stimuli responsive DDSs; including smart DDSs for PDT, PTT and light-triggered release DDSs, (B) magnetic field responsive DDSs; including MNPs for magnetic field controlled targeting and MNPs for hyperthermia.

Herein, we reviewed the recent researches on stimuli-responsive DDSs for treatment of head and neck cancer. The DDSs could respond to the tumor TME of HNC such as acidic pH, ROS and matrix metalloproteinases (MMPs), reducing environment (Figure 1). Also, the DDSs that could be triggered by external physical stimuli have been reviewed (Figure 2). Other recent advances on the multistimuli responsive DDSs with more biofunctions have been summarized and prospected as well.

## Internal-stimuli responsive DDSs

### pH-responsive DDSs

pH-responsive DDSs are among the most reported intelligent DDSs in HNC therapy considering the acidic tumor microenvironment (TME) distinguishing from normal tissue (Figure 1(A)). Hypoxia condition and high rate of anaerobic glycolysis in the TME could cause increasing production of acidic metabolites by tumor cells in the extracellular matrix and develop a mildly acidic microenvironment, the pH of which drop to around 6.5, while the pH of normal tissues is about 7.4. The pH of cytoplasm or organelles are found even lower, such as endosomes (pH 5–6) and lysosomes (pH 4–5) (Matsumura & Maeda, 1986; Kanamala et al., 2016). The acidic TME could promote the extracellular-matrix remodeling and stimulates acid-activated proteases for increased local invasion, metastasis and resistance to treatments (Gillies et al., 2004; Rofstad et al., 2006). Yet, the pH discrepancy could also provide a platform for pH responsive DDSs. In recent years, increasing number of relative researches has been reported for the treatment of HNC to improve the efficiency and specificity of drug delivery (Karimi et al., 2016). Table 1 lists recent reports of pH-triggered DDSs for HNC. Various kinds of carriers including nanoparticles, micelles, hydrogels and so on have been applied and functionalized for pH-responsive drug delivery.

**Table 1.** pH-responsive DDSs for HNC therapy.

Effective pH	Carriers^a^	Drugs^a^	Tested cell^b^/model	Ligand/receptor	Remarks	Ref
6.8	OA-b-poly(Lys-g-DMA)-CD Nano-vehicle	DOX	KB		Increased doxorubicin uptake and higher tumor cytotoxicity	Lee, Lee, Kim, et al. (2014)
6.0	Starch–(GC–DEAP) Nanogel	D-(KLAKLAK)_2_ peptide	KB		Increased tumor cell ablation	Kim et al. (2017)
5.0	GQDs	CDDP	HSC3, SCC4, and CAL-27		Combating hypoxia-induced chemoresistance and decreased toxicity of cddp	Wei et al. (2018)
4.0/5.0/6.0	PAA-ACL-MSN	DOX	HNE-1		Great biocompatibility, better therapeutic efficacy and low toxicity	Chen et al. (2014)
5.0	MG⊂ Liposome	DOX	KB	FA receptor	Enhanced specificity of drug release and less side effect	Liu, Gao, et al. (2014)
5.5	MSN-phSA	Am D	KB	FA receptor	Enhanced cellular drug uptake and selective drug release	Datz et al. (2016)
5.0	HMSN-GM-CS-FA	PA and DOX	KB	FA receptor	Synergistic antitumor efficacy of photothermal-, photodynamic- and chemotherapy	Yan et al. (2020)
5/6.5	UCNP-Al-NH-PEG-NH-FA	DOX	KB	FA receptor	A promising NIR imaging agent and an efficient anticancer drug	Tawfik et al. (2018)
5.3	TW-80-SeNPs	5Fu and Cet	CNE	EGFR	Excellent MR imaging and tumor inhibition	Huang, Huang, et al. (2019)
5.5/6.5	bis-amino-terminated PEG	DOX	CAL-27 and SCC-25	HN-1 peptide	Higher selective cytotoxicity and enhanced tumor inhibition	Wang, Wan, et al. (2017)
5.0	HPAH micelle	DOX and autophagy inhibitor (LY294002)	HN-6 and CAL-27		Increased tumor sensitivity to DOX and higher proliferation inhibition of tumor cell	Saiyin et al. (2014)
5.0	HPAE NPs	NaAsO_2_ and MTH1 inhibitor (TH287)	CAL-27		More effective inhibition of tumor cell proliferation	Li et al. (2017)
5.0	PEOz-PLA	DOX and P-gp inhibitor (TPGS1000)	KB and KBv		Desired tumor targeting, enhanced uptake and promising vehicle for overcoming MDR	Zhao et al. (2015)
6.5	MOFs@gel	DOX and Cel	KB and SCC-9		Inhibiting the growth of tumor	Tan et al. (2020)
6.5	acetalated dextran polymer	Oxygen Nano-bubbles	CNE2		Combating the hypoxia-induced resistance	Song et al. (2019)
5.5	PEGylated LPH	DTX and P-gp inhibitor (TPGS)	SCC-7		Inducing tumor cell apoptosis and reducing the tumor size	Kim et al. (2015)

^a^The carriers and drugs are shown in following abbreviations: OA: oleic acid; poly(Lys): Poly(_L_-lysine); DMA: 2,3-dimethylmaleic acid; CD: β-cyclodextrin; DOX: doxorubicin; GC: glycol chitosan; DEAP: 3-diethylaminopropylamine; GQDs: graphene quantum dots; CDDP: cisplatin; FA: folic acid; hyd: hydrazine; PAA: poly(acrylic acid) homopolymer; ACL: acid cleavable linker; MSN: mesoporous silica nanoparticle; MG: Malachite green carbinol base; phSA: benzene sulfonamide group; Am D: Actinomycin D; HMSN: hollow mesoporous silica nanoparticle; CS: chitosan; PA: pheophorbide a; UCNP: upconversion nanoparticles; Al: Alginate; PEG: Poly(ethylene glycol); TW-80: Tween-80; Se: selenium; NPs: nanoparticles; 5Fu: 5-fluorouracil; Cet: cetuximab; HPAH: hyperbranched polyacylhydrazone; HPAE: hyperbranched poly(amine-ester); MTH1: MutT homolog 1; PEOz: poly(2-ethyl-2-oxazoline); PLA: poly(_D,L_-lactide); P-gp, P-glycoprotein; MOFs: metal-organic frameworks; Cel: celecoxib; LPH: lipid polymer hybrid; DTX: docetaxel.

^b^Cell lines: KB cell, HNE-1, CNE, CNE2, human nasopharyngeal carcinoma cell lines；HSC3, HN-6, SCC-4, SCC-7, SCC-9, SCC-25, CAL-27, Human oral squamous cell carcinoma cell lines; KBv, KB’s multidrug resistance counterpart.

Two types of pH-sensitive DDSs applied in HNC therapy (Taghizadeh et al., 2015; Li, Yang, et al., 2019). One is that the polymeric systems with ionizable groups undergo conformational or dissolution properties changes in response to altered pH values, thus leading to controlled drug release, which is more commonly applied in pH-sensitive DDSs for HNC. For example, Lee, Lee, Kim, et al. (2014) reported a kind of pH-responsive nano-vehicles with dimethylmaleic acid moieties which could be protonated and degraded at a slightly acidic pH. The DDS showed accelerated release of doxorubicin in pH 6.8 and significantly inhibit the viability of KB cells. Kim et al. (Kim et al., 2017) developed polysaccharidic nanogels with pH-responsive 3-diethylaminopropylamine for the delivery of proapoptotic d-(KLAKLAK)2 peptide. The DDS showed significant a significant increase in peptide release at acidic pH and KB cell ablation. Wei et al. (2018) synthesized a polyethylene glycol (PEG) modified graphene quantum dots-based nanocomposite loaded with Pt, which demonstrated pH-activated drug release and great tumor accumulation.

The other type of pH-responsive DDSs is that the acid-sensitive bonds attached to the polymer backbone may disintegrate in acidic TME, which could trigger the release of anticancer drug molecules. Chen et al. (2014) constructed a mesoporous silica nano-container with an acid cleavable linker for intracellular controlled release. With the degradation of the linker at lysosomal pH, the DDS showed controlled release of doxorubicin, leading to significant cytotoxicity on the HNE-1 cells. Saiyin et al. (2014) developed a pH-responsive nanomicelle for the treatment of oral squamous cell carcinoma (OSCC). The chemotherapy drug doxorubicin was conjugated onto the carriers by acylhydrazone linkages which were cleavable in acidic condition, leading to drug release leading to a significant proliferation inhibition of tumor cells.

Furthermore, positive targeting pH-responsive DDSs have also been fabricated in many studies. Various kinds of ligands have been conjugated DDSs have already been reported. Folic acid (FA) is widely utilized to functionalize the carriers since folate receptor (FR) have been reported highly expressed in many cancer cells but rarely in normal cells (Narmani et al., 2019). Therefore, FA conjugated DDSs such as carbon nanotubes (Wen et al., 2013), liposomes (Liu, Gao, et al., 2014), mesoporous silica nanoparticles (Datz et al., 2016; Yan et al., 2020) has been developed in recent years. For instance, Liu, Gao, et al. (2014) demonstrated FA conjugated malachite green carbinol-based liposome with highly efficient cellular and pH-sensitive doxorubicin release. Datz et al. (2016) synthesized pH-responsive mesoporous silica nanoparticles combined with FA for KB cell targeting and delivery of actinomycin D. Other kinds of pH-responsive DDSs such as anti-EGFR nanoparticles and HN-1 peptide grafted nanoparticles for HNC tumor cells accumulation has also show great potential of specific drug delivery (Wang, Wan, et al., 2017; Huang, Huang, et al., 2019). Other targeting agents like antibodies, nucleic acids, receptor ligands, etc. are also worth investigating in the future development of pH-responsive DDSs (Salahpour Anarjan, 2019).

The multidrug resistance (MDR) of HNC tumor cells has become one of the major problems of chemotherapy (Zhou et al., 2018). Beside of more specific drug delivery, several other strategies have also been applied in the pH-responsive DDSs research. Small molecules inhibitors like autophagy inhibitors, MTH1 inhibitors, P-glycoprotein inhibitors, etc. have been reported co-loading with chemotherapeutic agents in the DDSs (Saiyin et al., 2014; Zhao et al., 2015; Li et al., 2017). Another co-loading therapy was reported by Tan et al. (2020). The metal–organic framework-based hydrogel was loaded with doxorubicin and a nonsteroidal anti-inflammatory drug, celecoxib, which demonstrated synergistic effect and outstanding tumor inhibition efficiency. Other adjuvant therapies such as oxygen delivery systems has also been developed. Song et al. (2019) designed oxygen nanobubbles with pH-responsive release of oxygen in the TME, which could help overcome the hypoxia-induced resistance.

In recent years multifunctional DDSs have drawn intense attention as well. With integrated targeted imaging and treatment agents, the DDSs could provide simultaneous diagnoses and treatment for HNC. Huang, Huang, et al. (2019) designed 5-fluorouracil and gadolinium chelates loaded selenium nanoparticles covered with EGFR-targeted drugs Cet. The DDS showed increased intracellular accumulation, acid activated drug release and excellent magnetic resonance imaging capability. Tawfik et al. (2018) reported pH-activated upconversion nanoparticles loaded with doxorubicin, which demonstrated enhanced luminescence intensity for NIR imaging and significantly inhibit the viability KB cancer cells. These researches demonstrated new strategies with the combination of diagnoses and treatment of HNC, which provides great potential for further research and clinical application.

### Matrix metalloproteinases (MMPs)-responsive DDSs

Matrix metalloproteinases (MMPs), a family of proteolytic enzymes which could degrade extracellular matrix protein. They are in low quantity and activity in normal situation but could be upregulated in many kinds of cancers including HNC, which have been considered closely related to cancer initiation, growth, and metastasis (Coussens et al., 2002; Gabriel et al., 2011). Especially, MMP-2, MMP-9, and Membrane type 1-MMP are most reported in the HNC research (Rosenthal & Matrisian, 2006; Chien et al., 2013; Kaomongkolgit, 2013; Virós et al., 2013). Thus, MMPs have been considered as biomarkers for cancer diagnostics and therapeutic targets. MMPs have also served as a type trigger for stimuli-responsive DDSs in recent years (Xiong & Gao, 2017; Yao et al., 2018).

In the research of HNC therapy, MMP-responsive DDSs for the local chemotherapy were reported recently. The DDSs carriers were usually incorporated with short linear peptide that could be degraded by MMPs (Figure 1(B)). For instance, Li, Tao, et al. (2019) designed doxorubicin micelles loading MMP-responsive hyaluronic acid (HA) hydrogels crosslinked by an MMP-2 degradable peptide for the treatment of OSCC. The hydrogel DDSs demonstrated MMP-2-responsive drug release and significant antitumor effect *in vitro* and *in vivo*. Damiani et al. (2017) fabricated a novel nano-ferritin complex, loaded with doxorubicin as well. The carrier was linked with a polypeptide shield and a short motif sequence for enhanced tumor accumulation and MMPs triggered drug delivery. The DDS showed enhances antitumor effects on several HNSCC cell lines. Many kinds of MMP-responsive DDSs based on carriers like liposomes, dendrimers, polymetric nanoparticles, etc. were reported for cancer therapy in recent years. Ligands incorporated DDSs and cell penetrating peptides linked DDSs for more specific intracellular tumor targeting were also investigated. MMP-responsive DDSs provide another strategy for intelligent drug delivery (Xiong & Gao, 2017; Yao et al., 2018). Therefore, more research on various kinds of MMP-responsive DDSs for HNC is of great potential for further study.

### Reactive oxygen species (ROS)-responsive DDSs

ROS, including hydrogen peroxide (H_2_O_2_), singlet oxygen (^1^O_2_), superoxide (O^2−^), and hydroxyl radicals (HO•), are oxygen-carrying active molecules produced by mammalian mitochondria, endoplasmic reticulum, and NADPH oxidase. In normal situation, ROS in tissue play an important role in modulating the of functions of proteins, regulating cell signaling, killing pathogens, etc. (Burgoyne et al., 2013; Shim & Xia, 2013; Li, Wen, Wen et al., 2016). However, unbalanced ROS levels could be related to many diseases. It has been reported that the ROS production is upregulated during the progression of several types of cancers including HNC (Qian et al., 2018). The mitochondrial dysfunction, abnormal metabolic process metabolism and genetic mutations could cause the accumulation of oxidized protein, DNA, and lipids (Trachootham et al., 2009). Therefore, the enhanced ROS production could serve as a potential trigger for intelligent drug delivery. ROS-responsive DDSs have gained increasing attention in the treatment for HNC. ROS-cleavable groups such as organoborane-based groups and sulfur-containing groups were incorporated to carriers for ROS-triggered drug delivery (Saravanakumar et al., 2017) (Figure 1(C)). Thioketal groups were mostly reported in the ROS-responsive DDSs for HNC. They are a kind of sulfur-containing linkers that could be degraded through oxidative means, thus leading to ROS-activated drug release. Li, Wen, You, et al. (2016) constructed doxorubicin and alpha-TOS loading ROS-triggered drug delivery copolymer nanoplatform linked with TK groups for effective oral cancer therapy. Integrin αvβ3 targeting groups were also incorporated for active drug delivery. The nanoparticles showed improved cellular uptake and ROS-triggered drug release. The release of alpha-TOS could help stimulate cellular ROS. The DDS demonstrated significant inhibition of oral tongue Cal27 cancer cell line and antitumor. Similarly, Wang, Wang, et al. (2019) developed TK linked nanoparticles modified with another commonly used target, FA, for selective entry into cancer cells and ROS-responsive drug delivery. With specific release of doxorubicin, the DDS induced enhanced apoptosis of OSCC cells, which demonstrated robust therapeutic performance. ROS-responsive DDSs has shown great potential in the treatment for HNC. Yet the research is still limited and further research on *in vivo* application and more controllable *in situ* ROS level is desirable. Some researchers have tried to combined ROS-responsive DDSs with light-responsive for enhance ROS level in TME which will be described below.

### Redox-responsive DDSs

As mentioned above, the increased generation of ROS induced high levels of oxidative stress in cancer cells, which also results in the compensatory upregulation of antioxidant. The reducing environment of tumors has been considered as an important target and biomarker of cancer, including HNC. The glutathione (GSH)/glutathione disulfide (GSSG) couple has been reported the most abundant redox couple in cancer cells. The concentration of GSH in tumor tissues has been found much higher than that in normal tissue (Bansal & Simon, 2018; Guo et al., 2018). The research on redox-responsive DDSs showed another novel strategy for the treatment of HNC. The redox-responsive linkers are incorporated in the carriers such as disulfide bonds and diselenide bonds, which could be cleavage by GSH, leading to controlled drug release (Huo et al., 2014) (Figure 1(D)). Sun et al. (2018) designed redox-responsive nanoscale micelles carrying doxorubicin for the treatment of laryngopharyngeal carcinoma. The micelles based on the heparosan (HEP) and deoxycholic acid conjugates (HSDs) could be internalized by human pharynx squamous carcinoma cell lines (FaDu cell) through clathrin-mediated endocytosis. With the link of disulfide bonds, the DDS demonstrated GSH-triggered drug release and significantly inhibited tumor cell growth. Fan, Wang, et al. (2020) reported a GSH-sensitive conjugated with disulfide bonds as well. The folate-targeted nanoparticles loaded with paclitaxel for the OSCC showed enhanced antitumor effect *in vitro* and *in vivo*. The research on redox-responsive DDSs is still limited right now. More research on multifunctional redox-responsive DDSs could be further conducted in the future.

## External-stimuli responsive DDSs

### Light-responsive DDSs

Light responsive DDSs has been widely studied in recent researches. Specific wavelength of light (such as ultraviolet, visible and infrared/near-infrared (NIR) fluorescence, etc.) represents as promising exogenous stimulus in the HNC tumor therapy, for its noninvasive feature and possibility of spatio-temporal control (Son et al., 2019; Wang et al., 2020). Especially near infrared (NIR) laser with better penetration and minor damage to tissues has been applied in quite many reports. The light responsive DDSs were mainly used in photodynamic therapy and photothermal therapy (Figure 2(A)). Some other light-trigged drug release systems were also developed (Gao et al., 2016; Civantos et al., 2018; Li et al., 2020).

Photodynamic therapy (PDT) has been recognized as an important therapy for HNC, with low side effect and little influence on the tissue structure and function of HNC patients (Meulemans et al., 2019). In the PDT process, photosensitizers (PSs) are activated by the light of specific wavelength and convert oxygen into singlet oxygen or other reactive oxygen species (ROS), leading to apoptosis, necrosis or autophagy of tumor cells. There are mainly two generations of PS, which is represented by photofrin and 5-aminolevulinic acid (Fan, Zhu, et al., 2020). However, the light-associated toxicity and hypoxia -induced drug resistance limits application of PDT (Agostinis et al., 2011). In recent years, increasing number of researchers have designed PS loading DDSs and proved these DDSs could help reduce light-associated toxicity and enhance the drug accumulation. Various kinds of nanocarriers have been developed for the more specific drug delivery and better biocompatibility, such as lipid-based nanoparticles, calcium phosphate nanoparticles, magnetic nanoparticles and other copolymer nano-carriers (Wang, Fei, et al., 2014; Haedicke et al., 2015; He et al., 2015; Hong et al., 2016).

Photothermal therapy (PTT) refers to induced hyperthermia in the targeted tissues under light irradiation for anti-neoplasm effect, which is considered as a promising strategy for HNC treatment (Curry et al., 2014; Fan, Zhu, et al., 2020). Optical absorbing materials have been used to convert light energy into heat under external illumination, among which gold nanoparticles (AuNPs) are most commonly applied. AuNPs have been reported to exhibit strong surface plasmon resonance (SPR) and thus convert light to heat efficiently (Link & El-Sayed, 2000). Gold-coated magnetic nanoparticles have also been applied in PTT researches. Moreover, some other gold loading DDSs have been developed as well. For example, Rao et al. Rao et al. (2018) reported a platelet-facilitated PTT (PLT–PTT). Gold nanorods (AuNRs) were loaded into PLTs for tumor sites targeting and enhanced PTT effect. Table 2 listed current reports of light-triggered DDSs for HNC therapy.

**Table 2.** Multifunctional light-responsive DDSs for HNC therapy.

DDS carriers	Anticancer agents^a^	Functions	Refs.
Fibronectin-mimetic peptide conjugated ION	Photosensitizer (Pc 4)	Tumor targeting; MRI imaging and PDT	Wang, Fei, et al. (2014)
Self-assembled core–shell nanoparticles	CDDP; pyrolipid	Chemotherapy and PDT	He et al. (2015)
Polyglutamate nanoparticles	ICG	PDT and PTT	Tarassoli et al. (2017)
Human serum albumin nanoparticles	ICG; CDDP	Tumor targeting; PDT, PTT and chemotherapy	Wang, Xie, et al. (2019)
RGDfK peptide conjugated calcium phosphate nanoparticles	Temoporfin; fluorescent dye molecule (DY682-NHS)	Tumor targeting; NIR fluorescence imaging and PDT	Haedicke et al. (2015)
Gold nanorods	siRNA oligos	Gene therapy and PTT	Wang, Yu, et al. (2016)
Evans blue derivative-functionalized gold nanorods	Hydroxycamptothecin	tumor targeting; PTT and chemotherapy	Wang et al. (2018)
PDPN antibody-conjugated gold nanoparticles	DOX	tumor targeting; PTT and chemotherapy	Liu et al. (2020)
Gold nanorods	Rose Bengal molecules	PDT and PTT	Wang, Wang, et al. (2014)
Au nanoring	Sulfonated aluminum phthalocyanines	PDT and PTT	Chu et al. (2016)

^a^The anticancer agents are shown in the following abbreviations: CDDP: cisplatin; ICG: indocyanine green; DOX: doxorubicin.

Light-stimuli released systems have also been demonstrated in recent DDSs researches. Copolymers with light sensitive and degradable groups could serve as carriers, loaded with various antitumor agents especially chemotherapy drugs. Xing et al. (2015) reported doxorubicin loading spiropyran-containing upconversion nanoparticles. The spiropyran amphiphilic group could be shift to a hydrophilic one with NIR exposure and detach from the copolymer carriers, leading to drug release. The DDS demonstrated active targeting to KB cells and NIR-triggered drug release.

Furthermore, multifunctional DDSs have shown great potential in the HNC treatment in recent years. In the research of light-responsive DDSs, the combination of PDT and PTT were mostly reported, of which the synergistic therapeutic effect were verified. Other therapy modalities like chemotherapy and gene therapy were also combined to the phototherapy (Agostinis et al., 2011). For instance, Song et al. (2020) designed chlorin e6 (Ce6) linked DDS co-loaded with cisplatin (CDDP) and metformin for Head and Neck Squamous Cell Carcinoma. The PS Ce6 showed laser-triggered PTT and PDT effect while CDDP and metformin served as the chemotherapeutic core. Nam et al. (2018) loaded polydopamine (PDA)-coated spiky gold nanoparticles (SGNPs) with a subtherapeutic dose of doxorubicin (DOX) for the chemo-photothermal therapy (chemo-PTT) or HNSCCs. The nanoparticles elicit robust antitumor T-cell immunity and exert strong therapeutic efficacy against tumors.

Indocyanine green (ICG), a kind of clinically used NIR-absorbing dye was reported able to convert light energy into heat due to internal conversion. Meanwhile, ICG was also reported cytotoxic effect on tumor cells with the generation of ROS (Wang, Xie, et al., 2017; Wang, Xiao, et al., 2019). Therefore, several studies on the multifunctional light-responsive DDSs loaded with ICG were reported. Tarassoli et al. (2017) developed copolymer nanoparticles loaded with ICG for NIR-trigged head and neck cancer therapy with improved drug accumulation and antitumor effect. Wang, Xie, et al. (2019) fabricated ICG-cisplatin nanoparticles for the treatment of OSCC, the coordination bond of which could be cleaved by a NIR-induced photothermal effect of ICG. The results showed that the nanoparticles showed synergistic effects of PTT/PDT and chemotherapy.

As mentioned before, gene therapy is considered as a promising HNC. Multifunctional light-responsive DDSs combined with gene therapy has been fabricated by several researches, among which RNA interference by the small-interfering RNA (siRNA) were mostly used strategy. Wang et al. (2016) developed gold nanorods linked with siRNA targeting Bcl-2 associated athanogene domain 3(BAG3) BAG3 could be induced by the heat shock response in PTT, leading to thermoresistance. The multifunctional nano-complex showed silence of the BAG3 expression in the Cal27 and improved PTT effect. Ma et al. (2017) reported Ce6 linked nanoparticle carrying Wnt-1 siRNA for oral cancer. Aberrant activated Wnt-1/β-catenin pathway was considered closely related to epithelial–mesenchymal transition (EMT) and progression of tumors. The DDS were proved to reduce the expression of Wnt-1, β-catenin and vimentin. The PDT effect was also enhanced. Ma et al. (2014) fabricated a star-shaped copolymer loaded with chemotherapeutic drugs docetaxel and MMP-9 shRNA plasmid for nasopharyngeal cancer therapy. The DDS showed light-enhanced gene transfection efficiency as the carrier was functionalized with porphyrin–arginine. The MMP-9 protein expression in HNE-1 cells was significantly reduced and the DDS showed synergetic antitumor effect compared to docetaxel or MMP-9 plasmid used alone.

Positive targeting was also reported in the light-responsive DDSs research. Ligands conjugated carriers were been fabricated for more specific drug delivery. For instance, Akbarzadeh et al. (2018) reported a FA-conjugated nanoparticle for the delivery of PS 5-aminolevulinic acid which demonstrated selective endocytosis into KB cells and increased effect of PDT. The SGNPs constructed by Nam et al. (2018) for chemo-photothermal therapy were also modified with low-density lipoprotein receptor (LDLR) for active tumor targeting.

Multifunctional light-responsive DDSs has been applied in the design of integrated materials of diagnosis and treatment for HNC as well. Near-infrared fluorescence dyes could be co-loaded in the DDSs for tumor imaging and tumor ablation. Haedicke et al. (2015) fabricated calcium phosphate nanoparticles conjugated with the PS temoporfin and fluorescent dye molecule DY682-NHS, which combined NIRF optical imaging and PDT therapy. Wang et al. (2018) developed Evans Blue derivative gold nanoparticles for imaging-guided cancer therapy. DDSs based on magnetic nanoparticles were also utilized as multifunctional tools for phototherapy and magnetic resonance imaging (MRI). For instance, Wang, Fei, et al. (2014) developed iron oxide nanoparticles loaded with PDT drug, Pc 4, showing improved MRI contrast and enhanced PDT efficacy. Further researches on multifunctional light-responsive DDSs are worth conducting.

### Magnetic field-responsive DDSs

The magnetic nanoparticle (MNPs)-based DDSs that could respond to external magnetic field have been extensively studied in diagnosis and treatment of the cancer (Figure 2(B)). They could serve as carriers for the delivery of antitumor agents controlled by external magnetic field, which could also be modified with various kinds of functional groups for stable drug loading, gene delivery, active tumor targeting and so on (Cardoso et al., 2018). In the treatment of HNC, MNPs have drawn increasing attention. Zhang et al. (2020) fabricated bleomycin loading hollow mesoporous magnetic nanoparticles for the treatment of HNC. The DDS were targeted to the focal area under the external magnetic field and demonstrated sustained drug release, which could induce the apoptosis of several kinds of head and neck cancer cell lines. Miao et al. (2014) have developed new PEI-modified Fe_3_O_4_ nanoparticles as a gene transfer vector to mediate transfection of OSCC by the targeting plasmids transport with an external magnetic field. The improved transfection efficiency and antitumor effect was verified *in vitro* and *in vivo*.

Furthermore, another important of MNPs is the heat generation induced by alternating magnetic field (LeBrun & Zhu, 2018). Therefore, MNPs could also serve as hyperthermia agents in the magnetic field-responsive DDSs, leading to antitumor effect. Su et al. (2019) designed the anti-CD44 antibody-modified superparamagnetic iron oxide nanoparticles targeting cancer stem cells of HNC. The nanoparticles could penetrate into the cells through the cell membrane, and inhibit grafted tumor growth in mice by hyperthermia induced in a magnetic field.

Other multifunctional magnetic field-responsive DDSs has also been reported with the development of the modification of MNPs, such as DDS with combination of diagnostics and treatment, ligand linked DDS and MNP-based multistimuli-responsive DDS which will be describe below.

## Dual and multi stimuli-responsive DDSs

As is showed above, great progress has been made in the stimuli-responsive DDSs for the treatment of HNC in recent years. Researches have also made great efforts to modify stimuli-responsive DDSs with multiple functions for more controllable drug delivery, better biocompatibility and less drug resistance. The carriers were modified with functional segments for active tumor targeting, better stability of DDS, more drug loading, etc. Various kinds of diagnostic and/or therapeutic agents were co-loaded to improve the treatment efficiency, overcome drug resistance, simplify the diagnosis and treatment procedures, and so on.

Moreover, another important strategy to further finetune drug release and enhance therapeutic efficacy of stimuli-responsive DDSs has gained great attention in recent years; that is, the development of novel dual or multi stimuli activated DDSs, which could respond to more than one internal and/or external trigger simultaneously or in sequence (Bhatnagar & Venuganti, 2015; Qiao et al., 2019). Table 3 lists recent research of emerging dual or multistimuli responsive DDSs for HNC therapy. DDSs with response to multiple TME triggers like pH, ROS, redox were reported with improved TME sensitivity. Zhang et al. (2016) prepared A pH and redox responsive mesoporous silica nanoparticles loaded with doxorubicin (DOX). The disulfide bond and H-bond could be degraded by GSH and acidic condition leading to increased drug release and inhibition of human tongue squamous cell carcinoma TCA8113 cell lines. Liu et al. (2019) constructed another kind of pH and redox responsive DDS for advanced nasopharyngeal carcinoma. The multifunctional DDS also showed folate-targeted codelivery of docetaxel (DOC) and tissue factor pathway inhibitor 2 TFPI2, which made cancer cells more sensitive to DOC and enhanced the antitumor effect compared with monochemotherapy.

**Table 3.** Dual/multiresponsive DDSs for HNC therapy.

Stimuli	Drug^a^	Target cell^b^/model	Mechanism	Remark	Ref
pH, redox	DOX	TCA8113	Sulfur–sulfur bonding of the molecule in SMIP decomposed by an acidic pH and GSH	Inhibited tumor cells growth, low cytotoxicity and great biocompatibility	Zhang et al. (2016)
pH, redox	DOC and TFPI-2	HNE-1	The hydrazone bond split by acidity and the disulfide linkages degraded in the cytosol increase the drug release	Induced cell apoptosis, reduced cell invasion and decreased the tumor size	Liu et al. (2019)
pH, light	Photosensitizer (C60)	KB	DMA blocks cleavage-activated drug release and cytotoxic singlet oxygen generation	Enhanced photodynamic tumor inhibition	Kim et al. (2014)
pH, light	Photosensitizer (ce6)	KB	DEAP protonation-mediated drug release and high-yield generation of cytotoxic singlet oxygen	Enhanced photodynamic tumor inhibition	Lee, Oh, et al. (2014)
Light, MMPs	DOX	SCC-15	MMP2-responsive accelerated degradation of the hydrogel	Increased antitumor efficacy and fluorescence imaging	Wang, Fu, et al. (2019)
Light, ROS	DOX	KB	ROS-responsive self-destruction and following H_2_O_2_-responsive drug release	Increased antitumor efficacy and decreased systemic toxicity	Wang, Zhai, et al. (2019)
Light, ROS	Photosensitizer (Cu_2−x_S)	HeLa tumor cell line-derived xenograft and HNSCC patient-derived xenograft	MnS-mediated O_2_ production overcomes hypoxia and NIR-triggered Cu_2−x_S leads to enhanced PD effect	Increased antitumor efficacy and magnetic resonance imaging	Huang, Deng, et al. (2019)
Light, ROS	DOX and photosensitizer (HP)	CAL-27	ROS-responsive thioketal bond cleavage leading to release of drug and photosensitizer for PDT	Increased antitumor efficacy	Shi et al. (2018)
Light, magnet	SiNC	KB	A magnetic field guide the release of photosensitizer at tumor for synergistic PDT/PTT	Increased antitumor efficacy and magnetic resonance imaging	Bhana et al. (2015)
Thermo, magnet	DOX	SQ20B	magnetic hyperthermia-mediated drug release	Increased antitumor efficacy and magnetic resonance imaging	Kim et al. (2013)
pH, magnet	BTZ	SCC-7	pH-responsive drug release and magnet-triggered hyperthermia	Increased antitumor efficacy	Sasikala et al. (2015)
pH, ROS, light	DOX	WSU-HN6	pH-triggered Fe^2+^ coordination bonds cleavage for drug release and photothermal therapy; H_2_O_2_-induced catalytic generation of hydroxyl radicals	Combined photothermal therapy, chemotherapy, and Fenton reaction-based tumor therapy	Jin et al. (2018)
pH, redox, light	Taxol	KB	pH-responsive boronate ester and DTT-responsive disulfide bonds for triggered drug release	Increased antitumor efficacy and confocal laser scanning microscopy imaging	Lee et al. (2014)

^a^The drugs are shown in the following abbreviations: DOX: doxorubicin; DOC: docetaxel; TFPI: tissue factor pathway inhibitor; ce6: chlorin e6; Cu_2−x_S: copper sulfide with numerous copper-deficient stoichiometries; HP: hematoporphyrin; SiNC: silicon 2,3-naphthalocyannie dihydroxide; BTZ: bortezomib.

^b^Cell lines: KB cell, HNE-1, Human nasopharyngeal carcinoma cell lines; SCC-7, SCC-15, SQ20B, TCA8113, WSU-HN6, CAL-27, human oral squamous cell carcinoma cell lines.

More researches were conducted on the intelligent DDSs with combined response to internal and external triggers, since physical exogenous stimuli could be more controllable and flexible. Such dual stimuli-responsive DDSs were widely applied in the phototherapy for HNC. PDT or PTT agents were loaded in pH or MMP sensitive drug carriers for TME controlled drug release and light triggered antitumor treatment (Tarassoli et al., 2017). Meanwhile, chemotherapy agents were also reported co-delivered for the improved chemophoto therapy (Wang, Hu, et al., 2019). Fluorescence dyes could also be co-loaded for simultaneous tumor detection. Furthermore, in view of the ROS generation triggered by light during the PDT for HNC, light and ROS dual responsive DDSs were considered promising in recent researches. Light-responsive agents were loaded in ROS-sensitive carriers, which lead to enhanced ROS generation for PDT and synergistical promotion of drug release combined with endogenous ROS. For instance, Shi et al. (2018) developed ROS-responsive nanoparticles co-loaded with doxorubicin (DOX) and photosensitizer hematoporphyrin for the treatment of oral tongue squamous cell carcinoma. The ROS-induced rupture of thioketal linkage caused the TME-responsive drug release. The DDSs showed significant antitumor effect after laser irradiation. Wang, Zhai, et al. (2019) synthesized ROS-sensitive DDS for OSCC, composed of the self-degrading polymeric carrier and photosensitizer chlorin e6 (Ce6). The significant PDT effect and a cascade reaction for the release of loaded DOX was verified in the research. Another form of light and ROS dual responsive DDS was reported by Huang, Deng, et al. (2019) The nanoplatform was integrated by plasmonic Cu2 − xS and magnetic manganese compounds. The Cu2 − xS core demonstrated PTT and PDT effect under NIR irradiation while the MnS shell showed ROS-responsive O2 production through Fenton-like pathways.

External magnetic field is another important physical stimulus applied in the research of multistimuli responsive DDSs. Magnetic nanoparticles have been widely studied as drug carriers in the diagnosis and treatment for HNC. In recent research, they were also reported combined with phototherapy, yielding magnetic field and light stimuli-responsive DDSs. For example, Bhana et al. (2015) presented iron oxide–gold nanostructures with magnetic field-guided drug delivery of NIR absorbing photosensitizer for targeting PDT and PTT, which significantly inhibited the KB cell viability. The researchers also mentioned the nanoparticle could be used for MRI and serve as a platform for tumor imaging and therapy. Moreover, considering the heat-generating properties of magnetic nanoparticles, iron oxide-based dual stimuli-responsive DDSs has also be reported for the hyperthermia therapy. Kim et al. (2013) reported multifunctional thermo and magnet responsive micelles encapsulated with superparamagnetic iron oxide nanoparticles. The DDS exhibited magnetic hyperthermia-mediated doxorubicin release and enhancement abilities of MRI contrast. With the integrin β4 antibody linked on the carriers, the DDS also demonstrated active targeting to squamous head and neck carcinoma cells. Sasikala et al. (2015) developed a smart magnetic nanoplatform for the pH-responsive delivery of anticancer drug Bortezomib (BTZ), which simultaneously showed hyperthermia effect triggered by the alternating magnetic field.

Beside of dual-stimuli responsive DDSs, other triple or multistimuli responsive DDSs have also been reported in recent years. Jin et al. (2018) conducted researches about core–satellite materials in response to pH, ROS and light stimuli for the treatment of OSCC. The nanoparticles showed NIR-induced PTT effect and pH-triggered doxorubicin (DOX) release. The ferrous iron linked on the nanoparticles could also catalyze the decomposition of ROS in tumor cells by a Fenton-like reaction. He et al. synthesized multistimuli responsive expansile nanogel DDS targeting head and neck tumor. The DDS exhibited intracellular release of photosensitizer PC4 triggered by acidic pH, high temperature, and elevated GSH and significant PDT effect under red light irradiation. Lee, Lee, In, et al. (2014) developed pH, redox and light responsive polymeric micelles for the delivery of anticancer drug Taxol and tumor cell imaging. The dual and multistimuli responsive DDSs exhibited great potential in the treatment of HNC, which deserve more research for improved material stability and synergistic antitumor effect.

## Conclusion and future perspectives

In this article, current progress of stimuli-responsive DDSs for the treatment of HNC were reviewed, which could respond to various kinds of triggers including the internal TME of HNC, external stimuli or multi stimuli. Their advantages and limitations were also summarized. It revealed that stimuli-responsive DDSs could be a promising nanomedicine platform for the anticancer drug delivery with improved therapeutic effect and systemic side effects over traditional drugs administration. Yet there are still many challenges which desire future studies are desirable. As mentioned before, although TME is quite different from normal, the condition is sometimes unstable and uncontrollable, which might limit the application of the internal stimuli-responsive DDSs. More strategies are desired to improve the sensitivity and controllability of stimuli-responsive DDSs. The combination of external stimuli responsive ability could be a useful option. Yet more research is still required for proper tissue penetration, more specific action range and less adverse effect on normal tissue around. Meanwhile, although the active targeting stimuli responsive DDSs has drawn growing attention in the HNC therapy, the research is still on the way to further exploration. Particularly, more preclinical tests are needed to investigated the antitumor effect and biocompatibility in the complex *in vivo* condition. Additionally, multifunctional and multi stimuli-responsive DDSs have showed great potential in theranostic applications, combined antitumor therapy, inhibition of MDR and other kinds of treatments for NHC as mention before. The fabrication of stimuli-responsive DDSs with optimized structure, simplified preparation process and improved *in vivo* therapeutic effect might become one of the development directions.

To sum up, stimuli-responsive DDSs have great prospect of clinical translation and more exploration, which provide a great alternative for NHC therapy. It is believed that with the development of fabrication techniques and further research, more stimuli-responsive DDSs will be applied for the treatment of HNC in clinic in the near future.
